# Ventricular Cerebrospinal Fluid Sampling in Pediatric Diffuse Midline Glioma Patients: Institutional Experience and Review of the Literature

**DOI:** 10.3389/fped.2020.556802

**Published:** 2020-10-27

**Authors:** Daphne Li, Wendy Stellpflug, Kathy Romanski, Maureen Kilgallon, Stacy Speck, Amanda M. Saratsis

**Affiliations:** ^1^Department of Neurological Surgery, Loyola University Medical Center, Maywood, IL, United States; ^2^Department of Surgery, Division of Pediatric Neurosurgery, Ann & Robert H. Lurie Children's Hospital, Chicago, IL, United States; ^3^Department of Neurological Surgery, Northwestern University Feinberg School of Medicine, Chicago, IL, United States; ^4^Department of Biochemistry and Molecular Genetics, Northwestern University Feinberg School of Medicine, Chicago, IL, United States

**Keywords:** pediatric diffuse midline glioma, hydrocephalus, ventricular access device, liquid biopsy, circulating tumor DNA

## Abstract

**Purpose:** Increasing evidence suggests that circulating biomarkers may serve diagnostic and longitudinal monitoring purposes in pediatric neuro-oncology. Mutant tumor DNA is detectable in the cerebrospinal fluid (CSF) of pediatric diffuse midline glioma (DMG) patients and quantity can reflect disease burden. CSF sampling (“liquid biopsy”) via a CSF access device could therefore play a role in DMG management. Therefore, we set to evaluate the incidence of hydrocephalus (HCP) in DMG patients, and to characterize ventricular reservoir placement and access practices.

**Methods:** A single institution retrospective review of DMG patients ≤21-years-old was performed (1984–2019). MEDLINE searches for reports of ventricular reservoir or shunt placement in DMG, and reservoir access for intraventricular chemotherapy (IVC) were performed.

**Results:** At our institution, 62.6% of DMG patients (67/108) required intervention for HCP: 19.4% provided transient CSF access (ETV alone *n* = 3, EVD *n* = 8, unspecified *n* = 2), and 80.6% permanent CSF access (ETV + reservoir *n* = 13, shunt *n* = 41). Further, 22/34 patients with initially transient CSF devices required conversion to a permanent device. Five devices were revised for malfunction, one for infection. Seventeen articles cited HCP in 22 to 100% of DMG patients. IVC administration was described in 632 patients (seven articles), with 42 infectious and 63 non-infectious complications.

**Conclusions:** Management of HCP is often necessary in children with DMG. Given the low rate of clinical risk associated with VAD placement and access, and the potential utility of longitudinal disease monitoring via CSF analysis, VAD placement could be considered in future clinical trials to guide DMG treatment.

## Introduction

Pediatric diffuse midline gliomas (DMGs), including thalamic and Diffuse Intrinsic Pontine Gliomas (DIPGs), and are highly morbid brain tumors. Children with DMG have a median survival of only 9 months, with a near 100% mortality despite treatment (1). Importantly, recurrent somatic missense mutations in histone H3 isoforms, resulting in H3K27M mutant proteins, are found in 80% of pediatric DMGs, and portend poorer prognosis and response to therapy (2–5). As a result, all gliomas harboring the H3K27M mutation are now considered high-grade, irrespective of histological features (6). In turn, clinical detection of this mutation is now critical to patient diagnosis and management, but tissue sampling from these deep seated tumors is not without risk, and is not feasible for longitudinal monitoring.

Importantly, tumor mutant DNA and proteins are detectable in cerebrospinal fluid (CSF) of children with DMG, with levels correlating with both CSF proximity to the tumor and changes in tumor volume in response to treatment (7–9). Circulating tumor DNA (ctDNA) levels in CSF from adult patients with brain tumors are also prognostic, demonstrating the potential clinical power of a “liquid biopsy” approach for clinical diagnosis and monitoring effects of therapy, including differentiating tumor recurrence from treatment response (10–16). Given the desperate need for more effective treatment for DMG, and the potential clinical utility of liquid biopsy for diagnosis and management, serial CSF sampling via lumbar puncture (LP) is now included in one pediatric clinical trial to identify clinical correlates of treatment response (17). However, since the concentration of ctDNA and proteins is greater in CSF collected from closer proximity to the tumor, serial ventricular CSF sampling may be of greater utility than LP. Therefore, in order to explore the incidence and feasibility of ventricular CSF sampling from children with diffuse midline glioma, we sought to determine the incidence and clinical outcomes of ventricular catheter placement and access in these patients at our institution, and as reported in the literature.

## Methods

The studies involving human participants were reviewed and approved by the Ann & Robert H. Lurie Children's Hospital of Chicago Institutional Review Board (IRB# 2005-12252). A retrospective review was conducted of the medical records of pediatric patients (≤21 years old) diagnosed with thalamic and brainstem glioma who received all or a portion of their neuro-oncologic treatment at the Ann & Robert H. Lurie Children's Hospital of Chicago from December 1984 to February 2019. Diagnosis, presenting symptoms, and neurosurgical interventions were evaluated, including the presence of hydrocephalus (HCP) with the need for CSF access or diversion during the course of treatment [lumbar puncture (LP), endoscopic third ventriculostomy (ETV), ventricular access device or reservoir (VAD), external ventricular drain (EVD), CSF or ventriculoperitoneal shunt (VPS)]. CSF access was categorized as transient if the patient underwent LP, ETV or EVD placement, or permanent if the patient received placement of a VAD or shunt.

A systematic MEDLINE search was also conducted using key words “pediatric” and “hydrocephalus,” “ommaya reservoir,” “ventriculoperitoneal shunt,” or “third ventriculostomy,” and “diffuse midline glioma,” “diffuse intrinsic pontine glioma,” “brainstem glioma,” or “thalamic glioma” (Supplementary Figure 1). A second systematic MEDLINE search was conducted using key words “pediatric ommaya reservoir” or “pediatric ventricular reservoir” and “intraventricular chemotherapy” (Supplementary Figure 2). Reports of tumors of the posterior fossa without brainstem involvement, tumors isolated to the pineal region or tectum, patients > 21 years of age, or lacking perioperative outcomes of cerebrospinal fluid access device placement were excluded, as well as those without full-text available for review in English. Reports were evaluated for VAD placement and access practices, interventions for hydrocephalus (HCP) management, and device-related complications.

## Results

### Institutional Cohort

A total of 3,521 pediatric brain and spine tumor patient records were available for retrospective review at our institution, with 108 diagnosed with DMG (Table 1). One of these patients' records was excluded because their case was reviewed for pathology purposes only (midline supratentorial thalamus, EVD). The remaining 107 patients received either all of their care at our institution, were seen in consultation for a second opinion (*n* = 17) or underwent biopsy and/or management of HCP at our institution prior to continuing care elsewhere (*n* = 2). Of these, 67 (62.6%) required a ventricular access procedure during their course of treatment (Table 2). A total of 13/67 children with DMG underwent transient CSF access procedures (19.4%, ETV alone *n* = 3, EVD *n* = 8, or unspecified *n* = 2), while 54/67 had permanent CSF access devices placed (80.6%, ETV + VAD *n* = 13, or CSF shunt *n* = 41). VADs were placed in these children for emergent ventricular access in case of ventriculostomy failure in combination with ETV (*n* = 13). An additional four DMG patients had clinical documentation of HCP but did not undergo intervention; three for unspecified reasons, and one because the family declined intervention. Of note, none of our DMG patients underwent VAD or shunt placement for intraventricular chemotherapy (IVC) administration, and none of our DMG patients underwent serial access of their VAD for any other indication.

**Table 1:** Case characteristics of 108 pediatric diffuse midline glioma (DMG) cases treated at our institution (1984–2019).

**Patient characteristics**	***N* (%) of cases identified**
**Gender**	
Male	56 (52.3%)
Female	51 (47.7%)
Average age at diagnosis (range)	10.2 +/- 4.5 y (1.2-19.90 y)
Average overall survival (range; n=66)	1.48 +/- 1.42 y (0.01-8.34 y)
**Anatomic Location**	
Midline supratentorial	54
Thalamus (bilateral)	52 (11)
Bilateral Thalamus	11
Hypothalamus	2
Brainstem	42
Pontine	30
Medullary	1
Pontine/Medullary	3
Multifocal DMG (hemispheric and/or infratentorial involvement)	11

*DMG, diffuse midline glioma; HGG, high grade glioma; y, years*.

**Table 2:** Rate of hydrocephalus (HCP) and/or cerebrospinal fluid (CSF) access in Diffuse Midline Glioma (DMG) patients at our institution.

**DMG Patient characteristics**	***n* (%) of DMG cases**
Patients with HCP and/or CSF access	71/107 (66.4%)
Patients CSF access/intervention	67/107 (62.6%)
Transient access	13/107 (12.1%)
ETV	3
EVD → VPS → EVD → ETV	1
EVD	8
ETV + reservoir → EVD	1
Unspecified	2
Permanent access	54/107 (50.4%)
Ventricular reservoirs	13
ETV → reservoir	1
EVD → reservoir	2
ETV + EVD → reservoir	1
VPS	41
ETV → VPS	7
ETV + reservoir → VPS	3
EVD → VPS	11
Subdural shunt → VPS	1
LPS	1
Patients with HCP without intervention	4

*CSF, cerebrospinal fluid; DMG, diffuse midline glioma; ETV, endoscopic third ventriculostomy; EVD, external ventricular drainage; HCP, hydrocephalus; LPS, lumboperitoneal shunt; VPS, ventriculoperitoneal shunt*.

Amongst the 67 DMG patients that required intervention for HCP, 25 (37.3%) were treated for HCP perioperatively, within 7 days of initial tumor biopsy or resection, and 13 (19.4%) underwent HCP treatment in a delayed fashion. Other patients did not undergo tumor resection or biopsy and were treated surgically for hydrocephalus alone. For most patients, CSF diversion was initially attempted without foreign body implantation, but ultimately conversion to an indwelling device was required due to persistent hydrocephalus. For example, ETV and/or EVD was attempted as the primary intervention in 50.7% of patients (*n* = 34/67; ETV alone *n* = 10, ETV with EVD *n* = 1, EVD alone *n* = 21, unspecified *n* = 2). Of the ten DMG patients who underwent initial ETV alone, eight eventually required an indwelling CSF access device (VAD *n* = 1, VPS *n* = 7). One ETV patient underwent a second ETV attempt, which subsequently failed and required VPS placement. Another patient with initial concurrent EVD and ETV ultimately required a VPS. 61.9% of patients with EVDs (*n* = 13/21) also required conversion to either a VAD (*n* = 2) or VPS (*n* = 11).

A total of eight DMG patients with HCP experienced multiple device-related complications (11.9%, Table 2). Five (7.5%) of these patients had non-infectious shunt malfunctions and device revisions: one subdural shunt was revised to a VPS, one patient required a single VPS revision, and three patients required two or more VPS revisions. Two patients with a VPS required a second ventricular catheter for entrapped ventricles. Only one infectious complication was identified (1.5%): this child had an EVD placed at the time of tumor resection that was converted to a VPS, and complicated by serratia odorifae infection two weeks later requiring externalization and intravenous antibiotics before reinternalization. This patient also suffered multiple non-infectious complications, including shunt malfunction requiring revision, undergoing shunt removal and ETV. Due to quality of and access to outside medical records, meaningful analysis of effects of different shunt hardware on outcomes was not feasible.

### Literature Review

Review of the literature yielded 18 articles describing 383 patients with glioma of the thalamus or brainstem, citing rates of HCP from 22 to 100% (overall 55.6%, *n* = 213/383), and intervention in 22–63% (overall 53.3%, *n* = 204/383) (Table 3) (18–35). Of these cases, there were 132 ventricular shunts (including 2 Torkildsen shunts) (22), 70 ETVs, and two EVDs that were ultimately weaned (31). Nine patients with evidence of HCP were untreated, with reports citing anatomic distortion from thalamic gliomas (*n* = 2 ETV deferred) (23), reasons unspecified, (36), patient refusal, or poor overall clinical condition (*n* = 6) (18, 22). Infectious complications were reported in association with placement of one Torkildsen shunt (0.01% of all shunts placed) (22). Non-infectious complications secondary to shunt placement included shunt malfunction [*n* = 4, one Torkildsen shunt converted to a VPS (22), one confirmed VPS malfunction requiring revision (18) and two suspected VPS malfunctions (25)], and contralateral subdural hematoma that resolved after placement of temporary subduroperitoneal shunts (*n* = 3) (31). Of note, there were four reported cases of extraneural metastases via CSF seeding through shunt systems (19, 28, 32, 34). These occurred most often after other evidence of tumor recurrence or progression, particularly with leptomeningeal dissemination. Reported ETV complications (*n* = 6, 8.5%) consisted of failure to effectively treat HCP requiring conversion to VPS (18, 23–25).

**Table 3:** Results of literature review for Cerebrospinal fluid (CSF) diversion procedures in children with diffuse midline gliomas (DMG) and hydrocephalus (HCP).

**References**	***N***	**Age**	**Tumor Diagnosis**	**Intervention for HCP**	**Complications from CSF diversion**
Jimenez-Jimenez et al. (19)	1	4 y F	Pontomesencephalic astrocytoma	VPS placement	Ascites 5m after diversion
Newton (34)	1	13 y M	Pontine GBM, leptomeningeal gliomatosis	VPS placement	Leptomeningeal gliomatosis with peritoneal and omental metastases
Masuzawa et al. (20)	8	2-16 y	Pontine glioma	5/8 (62.5%) required shunting/drainage	0
Zagzag et al. (21)	7	Median 1-8 y	Brainstem glioma - PNET	VPS in 100% - 5 (71.4%) at diagnosis, 2 (28.6%) at recurrence/progression	0
Amano et al. (22)	18	3-15 y	Pontine glioma	2 Torkildsen shunts 7 VPS 3 ETV 4 patients refused intervention for HCP	2 Torkildsen shunts (1 required VPS, 1 fatal infection)
Ray et al. (23)	14	Median 9.8 y	Brainstem glioma	14 ETV	3 (required VPS)
	6		Thalamic glioma	4 ETV 2 patients untreated for poor anatomy	0
Klimo and Goumnerova (24)	9	Median 5.8 y	DIPG	9 ETV	1 (required VPS)
Puget et al. (26)	69	9.5 +/-4.4 y mean (0-16 y) unilateral; mean 9.3 y (3-13 y) thalamic; mean 9.6 y bilateral	Thalamic tumors−54 unilateral, 6 thalamopeduncular, 9 bilateral	34/54 (63%) unilateral tumors required fluid diversion−24 VPS (71%), 10 ETV (29%) 6/6 thalamopeduncular (100%) required diversion−3 VPS, 3 ETV 6/9 bilateral (66.7%) had HCP and 5 (56%) required diversion with VPS	0
Raja and Adada (35)	1	15 y	DIPG	1 ETV	0
Roujeau et al. (25)	51	Median 7 y (3.1-16.2 y)	Diffuse brainstem glioma	2 ETV 9 VPS	1 ETV failure (required VPS) 2/9 VPS with dysfunction, no evidence on reoperation
Garzon et al. (27)	65	Median 8 y (13.9m to 17.6 y)	Brainstem glioma (22 DIPGs)	40/65 (61.5%) patients underwent surgery for HCP−33 VPS, 7 ETV; 24 before tumor resection 10/22 DIPG patients−7 VPS, 3 ETV	0
Barajas Jr. et al. (28)	1	6 y F	Pontine anaplastic astrocytoma	VPS at presentation	HCP and abdominal metastases 23m post-op
Fukuoka et al. (29)	1	8 y M	Brainstem anaplastic oligoastrocytoma (H3.3K27M)	None	N/A
	1	2 y F	Pontine oligoastrocytoma, Obstructive HCP	VPS placement 3d after craniotomy for subtotal tumor resection	0
Kobayashi and Ogiwara (33)	6	6.1 y mean	Brainstem glioma (5 DIPG)	6 ETV	0
Cinalli et al. (31)	27	9.53 y mean (3-17 y)	Thalamic tumors−9 unilateral, 16 thalamopeduncular, 2 bilateral	3 EVD (removed in 1, converted to VPS in 1, outcome not specified in 1) 6 ETV 2 septostomy + VPS (bilateral tumors) 1 VPS implanted later for delayed HCP	3 (subdural collections requiring diversion)
Gelder et al. (32)	1	4 y and 9m F	DIPG (H3.3K27M)	VPS at presentation	Nodules along shunt tract 10m after diversion
Guida et al. (30)	2	Median 5 y	DIPG	2 ETV	0
Giussani et al. (18)	9	Mean 6.29 +/- 3.4 y	DIPG	28 VPS 3 ETV 2 untreated (poor clinical status)	1 VPS malfunction (revised) 1 ETV failure (required VPS)

*CSF, cerebrospinal fluid; d, day; ETV, endoscopic third ventriculostomy; EVD, external ventricular drain; HCP, hydrocephalus; HGG, high grade glioma; ICP, intracranial pressure; m, month; N/A, not applicable; OS, overall survival; PNET, primitive neuroectodermal tumor; VPS, ventriculoperitoneal shunt; y, year*.

Review of the literature also yielded six articles describing placement of 492 VADs for IVC delivery in 483 patients with brain tumors. There were no reports of VAD placement for IVC administration in DMG patients. CSF reservoirs were in place for up to 70.3 months in some cases, with reports of up to 280 IVC instillations per device (Table 4). 42 infectious (8.5%) and 46 (9.3%) non-infectious reservoir related complications were reported, some occurring in the same patient (Table 4) (37–42). The most common non-infectious reservoir-related complications included catheter migration and/or malfunction (*n* = 14), and CSF leak and/or wound dehiscence (*n* = 18). Nine ventricular reservoirs were eventually converted to VPS for treatment of HCP.

**Table 4:** Results of literature review for clinical outcomes of serial access of Cerebrospinal fluid (CSF) access devices in pediatric brain tumor patients.

**References**	***N***	**Age**	**Indication**	**Duration of Device Use/Presence**	**Reservoir-related Complications**		**Interventions**
					**Infectious**	**Non-Infectious**	
Mead et al. (36)	91	< =18 y	IVC	Median 316d per device	1 (3%)	Not specified	NS
Palejwala et al. (39)	1	3 y	IVC and HCP	12 months	0	0	0
Peyrl et al. (38)	98	3 m to 21 y	IVC	5,472 IVC instillations (median 36 deliveries; 2-280/reservoir)	1 (1%)	1 catheter kink at burr hole 1 delayed catheter malposition due to shrinking of ventricles 1 delayed catheter disconnection	1 infection requiring removal despite antibiotics 3 return to OR for catheter revision
Pompe et al. (40)	211	Median 5.72 y (0-20.31 y)	IVC	2-4 cycles of 4-12 IVC instillations per device per IVC trial protocol	32 (71% staph: 41% staph. epidermidis, 18% staph. aureus)	13 malfunction 6 misplacement 11 CSF leak 1 unexplained removal 1 failed first implantation	Infection–revised in 25 Malfunction or misplacement–removed in 11 Failed first implantation–successful Rickham reservoir placed stereotactically
Gerber et al. (37)	20 (31 OR)	Median 3.3 y	IVC	16868 reservoir d total (155d median per device; 2-2871d) and 461 IVC instillations (median 9 per device; 0-37)	7 (CoNS−4 related to surgery, 3 related to reservoir use)	6 wound dehiscence 3 catheter misplacement 1 CSF leak	17 removals
Ozerov et al. (41)	60	Mean 7.6 +/- 4.9 y	IVC	Median follow up 8.5m (0-70.3m)	1 (1.9%)	1 parenchymal cyst around catheter	1 infected catheter removed 16d after implantation 1 catheter removal 60d after with cyst resolution

*CoNS, coagulase-negative staphylococcus; d, day; HCP, hydrocephalus; IVC, intraventricular chemotherapy; m, month; y, year*.

Of note, among all VAD placement procedures reported in this review, intra-operative catheter misplacement or failure of reservoir placement occurred in seven instances (41, 42). In addition, there was one instance of a catheter-associated cyst, which resolved after removal of the catheter (42).

## Discussion

Brain tumors are the most common solid tumor of childhood and leading cause of cancer death in children. Of these, DMGs have the highest mortality, with no improvement in clinical outcomes despite over 40 years of clinical trials (1). Recent advances in stereotactic surgical technology now enable surgeons to perform tumor biopsy, providing tissue for histologic and molecular analysis. Importantly, the recent discovery of a high rate of histone H3 mutation in DMG, and oncogenic downstream effects, has led to new therapeutic strategies and clinical trials (1, 2). As a result, tissue analysis for histone H3 mutations is now routinely performed for molecular diagnosis (5). In turn, multiple studies have evaluated biomarkers of various central nervous system tumors and their utility in diagnosis or longitudinal monitoring of response to treatment, including proteins and nucleic acids in the blood and CSF (43–45). While repeat tumor biopsy is often not clinically feasible, sampling of these liquid specimens to monitor Histone H3 mutant ctDNA or other potential disease biomarkers could prove valuable in guiding management. Importantly, sufficient tumor DNA and protein can be extracted from CSF retrieved from the lateral ventricle of a patient with thalamic or brainstem glioma, and ctDNA levels have been shown to correlate with changes in tumor size in response to targeted therapy (7–9). While children with brain tumors often undergo LP to assess disease dissemination or as part of chemotherapeutic protocols for intrathecal drug delivery, LP can be painful and dangerous in patients with an intracranial mass lesion. In addition, multiple studies have demonstrated that ventricular CSF is superior to lumbar for ctDNA sequencing and quantification, due to a higher concentration of ctDNA with closer proximity to tumor (7–9). Therefore, lateral or fourth ventricular device placement for CSF sampling may serve as a safer and more effective method for liquid biopsy compared to LP.

While ventricular device placement requires a neurosurgical intervention with foreign body implantation, retrospective review of our institutional cohort of DMG patients and the literature shows that over half already undergo intervention for HCP during their clinical course (62.6%, *n* = 67/107 and 53.2%, *n* = 204/383, respectively). While HCP may be treated in select cases with favorable anatomy and CSF dynamics via ETV to avoid foreign body implantation, 81.8% (*n* = 9/11) of DMG patients in our institutional cohort treated with ETV ultimately required permanent device placement. In addition, of the 21 patients in our cohort with an EVD, 62% (*n* = 13) required conversion to permanent devices. These data further demonstrate that ventricular access devices (VAD or shunt) are often necessary in this patient population: a clinical trial testing ventricular placement vs. ETV in DMG patients with hydrocephalus incorporating the ETV success score would therefore be prudent (46). Further study to determine measurable patient or tumor characteristics that can predict which patients will require permanent CSF diversion or suffer a complication, is also warranted. For now, current rates of ventricular device placement (VAD or shunt) could facilitate both CSF diversion and CSF sampling in the majority of these patients.

Importantly, the morbidity associated with ventricular catheter placement in this patient population is also very low. The most common non-infectious complications reported in the literature are device malfunction, misplacement of the catheter, and wound dehiscence with associated CSF leak. Importantly, there were no cases of DMG patients with wound dehiscence and CSF leak after VPS, ETV, or EVD placement in our patient cohort. Device malfunction was reported in only 2.8% of VADs (*n* = 14/492) and 2.9% of shunts (*n* = 3/104) in the literature, while we observed shunt malfunction in 12.1% of our DMG patients (*n* = 5/41). Complications related to ventricular catheter placement itself (2%; *n* = 10/492 in the literature) may be minimized with use of endoscopy, stereotactic neuronavigation, and intra-operative confirmation of CSF access through the device (39, 42).

Indeed, ventricular catheter placement is not without significant clinical risk. We found that only 64% of our DMG patients required ventricular access for HCP management, and do not recommend children undergo surgeries that are not clinically indicated: whether a liquid biopsy will provide information that will benefit patients in the form of decreased need for surgery or increased survival remains to be determined in clinical trials. However, given the potential for CSF studies to guide therapy in the future, we sought to evaluate the potential risks of VAD placement and access in DMG patients without HCP *for the sole purpose of CSF sampling*. We therefore looked to a patient population without HCP that undergoes ventricular catheter placement and serial device access for interventricular chemotherapy (IVC). Our review revealed a slightly higher rate of infectious (8.5%) and non-infectious (9.3%) complications associated with serial VAD access for IVC administration, relative to device placement alone. Indeed, it is likely that repeated mechanical access of these devices, in the setting of local and systemic effects of the chemotherapy, increases complication risk in these patients, underscoring the need for a stringent protocol for sterile device access. Martinez et al. found a high degree of variability in techniques used by neurosurgeons to access ventricular reservoirs or shunts, including skin cleansing solutions, and use of sterile draping (47). Importantly, multiple studies note that standardized perioperative antibiotics and local antiseptics decrease rates of infectious complications of serial VAD access (47, 48), suggesting the relative safety of VAD access in DMG patients for liquid biopsy purposes. In line with this, we, and others, have found that implementing a standardized protocol for VAD access, and increasing practitioner education and awareness of this protocols at all levels of the patient care team, can significantly reduce the risks of VAD associated infection (49–51). For example, a retrospective review of a cohort of 37 neonates with post-hemorrhagic HCP who underwent serial device taps at our institution using a stringent sterile access protocol revealed no infections after a total of 630 cumulative taps (average 17 taps per reservoir; range 0–83) across 10,420 collective indwelling reservoir days (average 282 per patient; range 6–3,700). Although reservoir access is usually performed by neurosurgeons, aspiration is also routinely performed by advanced practice nurses at our institution, and studies have documented low rates of complications when aspiration was performed either neurosurgery or neonatology teams (50, 52).

Of note, we could not identify any cases of VAD placement for IVC for DMG patients, either at our institution or in the literature, so should use caution when extrapolating these findings. Indeed, it is important to note that none of our DMG patients underwent serial access of their VAD, neither for IVC administration or other indications, so we cannot comment on whether this would have resulted in increased infection rates. Interestingly, there are reports that VAD placement for IVC administration into the fourth ventricle is feasible, with a low rate of complications (53, 54). Since the concentration of mutant ctDNA in CSF is greater with closer proximity to tumor, placement of a fourth ventricular catheter for longitudinal monitoring of liquid biomarkers may be of particular consideration for DIPG patients. As mentioned above, however, adequate ctDNA can be isolated from a lateral ventricular catheter (8, 9), so a neurosurgeon's best judgement should always be exercised when considering catheter placement.

## Limitations

Due to the retrospective nature of this study, limitations include inconsistent or incomplete documentation of patient clinical data. Some patients were treated before the implementation of the queried electronic medical record, and some received portions of their care at multiple institutions. The decision to include patients who were seen only in consultation for a second opinion was made in order to better examine the incidence of HCP in a large sample size. Exclusion of these patients eliminates the one documented infectious complication in our cohort, as well as two patients who required revisions for shunt malfunction. It is possible that the two patients who continued their care at outside institutions did have revisions or infectious complications that were not captured in our retrospective review. Best efforts were made to reconcile available data with any additional details available on review of patient electronic medical records. Despite these limitations, the authors believe that this literature review and retrospective cohort analysis accurately demonstrates indications, incidence, and outcomes of HCP management and ventricular reservoir placement in the examined patient populations.

## Conclusion

A review of the literature and our own institutional practice reveals that over half of pediatric DMG patients require a CSF diversion device for management of HCP. Additionally, ventricular catheter placement is technically feasible in children with DMG, with a low rate of associated complications when a standardized protocol for device access is utilized. Liquid biopsy from these patients could potentially aid in stratification to targeted therapies and disease monitoring. As a result, CSF sampling could be considered in future clinical trials as part of a liquid biopsy approach to guide clinical management and measure treatment response, with the goal to ultimately improve clinical outcomes for these devastating pediatric brain tumors.

## Data Availability Statement

All datasets presented in this study are included in the article/ Supplementary Material.

## Ethics Statement

The studies involving human participants were reviewed and approved by the Ann & Robert H. Lurie Children's Hospital of Chicago Institutional Review Board (IRB# 2005-12252).

## Author Contributions

DL is the first author having performed the majority data analysis, systematic review and writing the manuscript. WS is a major contributor in data collection. KR, MK, and SS participated in the review of the manuscript. AS is the principal investigator having contributed to the majority of the conception and review of the manuscript. All authors contributed to the article and approved the submitted version.

## Conflict of Interest

The authors declare that the research was conducted in the absence of any commercial or financial relationships that could be construed as a potential conflict of interest.
