# Supplementary figures and images for: Ventricular Cerebrospinal Fluid Sampling in Pediatric Diffuse Midline Glioma Patients: Institutional Experience and Review of the Literature
Source: Front Pediatr. 2020 Oct 27;8:556802. doi: 10.3389/fped.2020.556802 (PMC7652764; doi:10.3389/fped.2020.556802)

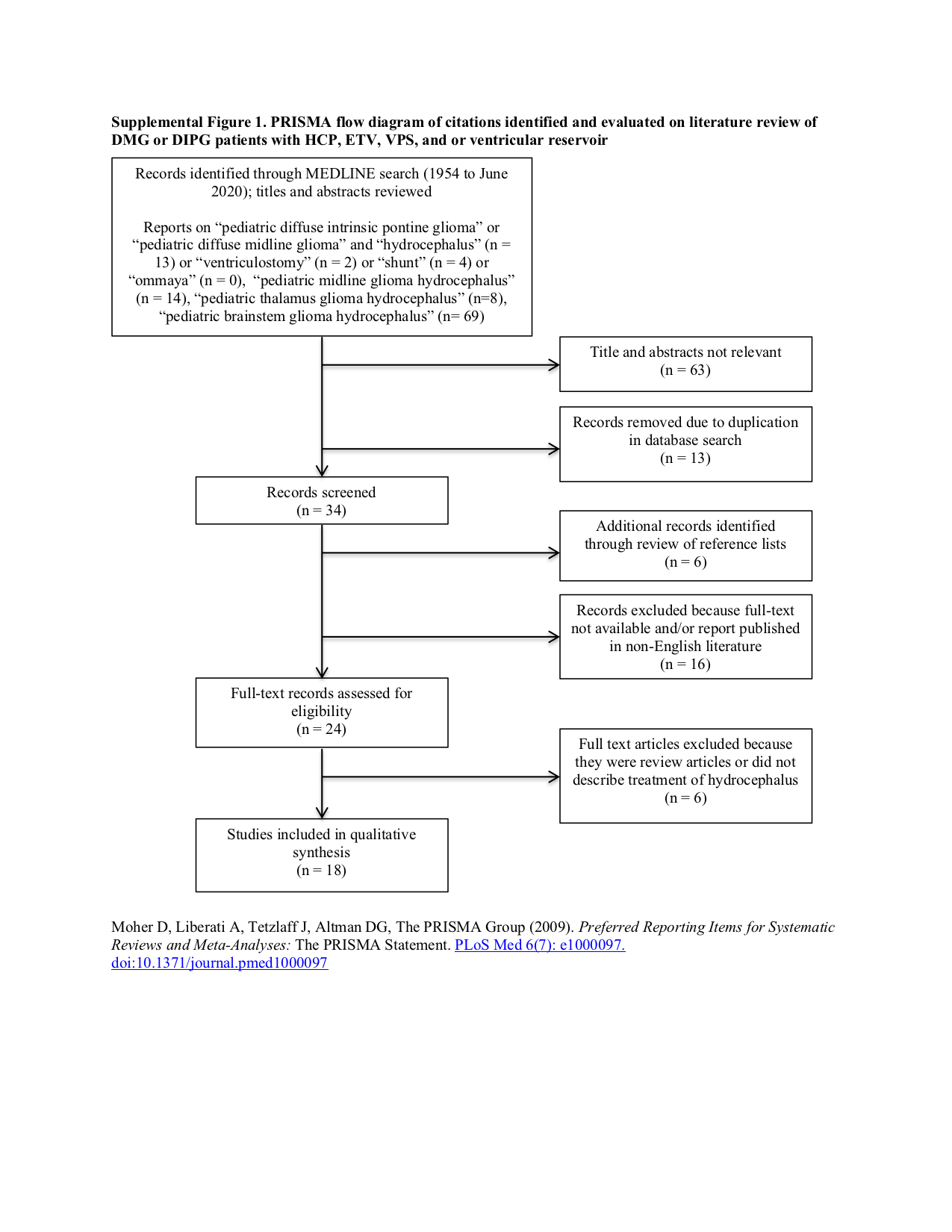

Supplement: Supplementary file 1 [file Image_1.TIFF]

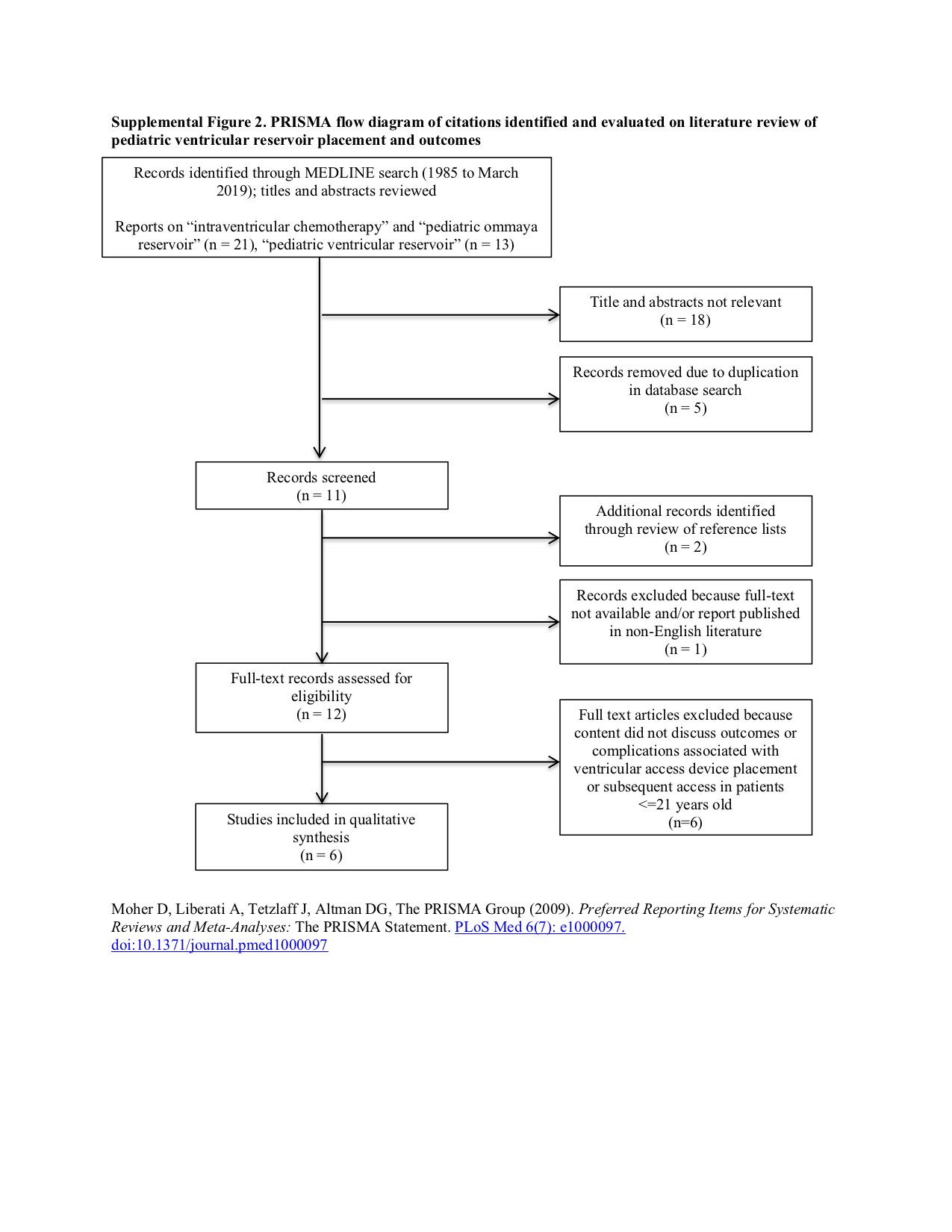

Supplement: Supplementary file 2 [file Image_2.TIFF]
